# Potential Effects of Nrf2 in Exercise Intervention of Neurotoxicity Caused by Methamphetamine Oxidative Stress

**DOI:** 10.1155/2022/4445734

**Published:** 2022-04-18

**Authors:** Lin Chen, Qin Ru, Qi Xiong, Jun Yang, Guodong Xu, Yuxiang Wu

**Affiliations:** ^1^School of Health and Physical Education, Jianghan University, Wuhan 430056, China; ^2^Wuhan Institutes of Biomedical Sciences, Jianghan University, Wuhan 430056, China; ^3^Library of Jianghan University, Jianghan University, Wuhan 430056, China

## Abstract

Methamphetamine can cause oxidative stress-centered lipid peroxidation, endoplasmic reticulum stress, mitochondrial dysfunction, excitatory neurotoxicity, and neuroinflammation and ultimately lead to nerve cell apoptosis, abnormal glial cell activation, and dysfunction of blood-brain barrier. Protecting nerve cells from oxidative destroy is a hopeful strategy for treating METH use disorder. Nrf2 is a major transcriptional regulator that activates the antioxidant, anti-inflammatory, and cell-protective gene expression through endogenous pathways that maintains cell REDOX homeostasis and is conducive to the survival of neurons. The Nrf2-mediated endogenous antioxidant pathway can also prevent neurodegenerative effects and functional defects caused by METH oxidative stress. Moderate exercise activates this endogenous antioxidant system, which involves in many diseases, including neurodegenerative diseases. Based on evidence from existing literature, we argue that appropriate exercise can play an endogenous antioxidant regulatory role in the Nrf2 signaling pathway to reduce a number of issues caused by METH-induced oxidative stress. However, more experimental evidence is needed to support this idea. In addition, further exploration is necessary about the different effects of various parameters of exercise intervention (such as exercise mode, time, and intensity) on the Nrf2 signaling pathway intervention. Whether there are synergistic effects between exercise and plant-derived Nrf2 activators is worth further investigation.

## 1. Introduction

Methamphetamine (METH) is one of the main kinds among amphetamine-type stimulants, which are the secondly most abused drug category in the world [1]. There is no effective drug treatment specifically for METH addiction [2]. Due to this, major challenges remain in treating METH use disorder (MUD) [2, 3]. The neurobiological properties of amphetamines are far more complex than the conventional understanding of monoaminergic modulators [3, 4]. Pharmacological treatment focused on regulating this monoaminergic pathway has largely failed [2, 5]. Novel pharmacological approaches are needed to find out for new therapeutic targets [5]. Therefore, novel treatments to manage MUD are urgently needed. It is essential to explore the intrinsic damage mechanism of MUD, at meanwhile, to note the key initiating role of oxidative stress in MUD. Nuclear factor erythroid 2-related factor 2 (Nrf2) is a crucial endogenous antioxidant factor [6]. It shows potential natural or pharmacological protective functions in a variety of diseases, including MUD [7]. However, identifying ways to activate endogenous antioxidant factors using nonpharmaceutical intervention methods to play a natural antioxidant role remains a problem for us to explore. Exercise intervention has become an emerging and promising approach [2, 8]. The purpose of our paper is to use the existing opinions of literature to explore whether exercise has the potential of actively mobilizing Nrf2 to play a critical role in the prevention and treatment of MUD-induced oxidative stress injury to the central nervous system.

## 2. The Important Effects of Oxidative Stress in the Neurotoxicity of METH

It has been demonstrated that the neurobiological mechanisms of MUD include but are not limited to oxidative stress, lipid peroxidation, endoplasmic reticulum stress, mitochondrial dysfunction, neurotoxic and excitotoxic effects, and intracellular pathways of neuroinflammation, which eventually lead to neuronal apoptosis [5]. METH is a psychostimulant, which mainly induces dopamine (DA), norepinephrine, and serotonin releasing [9]. The METH poisoning process is closely related to DA release induction. In general, activation of neuron promotes the DA release into synapses [10]. DA transporters (DATs) remove DA from synapses, and the vesicular monoamine transporter 2 (VMAT2) transports cytoplasmic DA into vesicles for storaging, releasing, and protection against reaction and oxidation consequences [11]. METH is extremely fat soluble and therefore crosses the blood-brain barrier into the brain easily [12]. It then enters the dopaminergic terminal via the DATs, because it is similar to DA [13]. Whereas METH easily leads to abnormal transport of DATs, which means that METH can increase levels of extracellular DA by stimulating abnormal secretion of DA and inhibiting reuptake of DA [11], METH also significantly increases the concentration of DA in the cytoplasm and synaptic cleft by impacting VMAT2 and improving DA release [14]. Extra DA is oxidized to semiquinone or quinone in the dopaminergic neuron terminus and synaptic clefts. In addition, a portion of DA metabolism, intermediated by monoamine oxidase (MAO), produces H_2_O_2_, a byproduct, or triggers an enzyme cascade reaction to produce rich reactive oxygen species (ROS) [15]. High levels of Glu induced by METH activate both N-methyl-D-aspartic acid receptors (NMDARs) and metabolic glutamate receptors (mGluRs) and lead to increased intracellular Ca^2+^ concentrations [16]. Abnormally increased calcium ion concentrations activate protein kinase, nitric oxide synthase, and phosphatase and promote NO production and other reactive nitrogen species (RNS) [17]. Overproduction of quinones or semiquinones, in addition to ROS and RNS, leads to endoplasmic reticulum (ER) stress and apoptotic pathways activation and induces ultimately neurotoxicity of METH, thereby promoting a suite of oxidative stress responses, including lipid peroxidation and protease activation, and ultimately triggering the cell death cascade [18]. Extracellular DA and DA-derived excitatory toxins also spread between interneurons and nonneurons in the brain. The synergistic effects of DA and GLUT-derived free radicals also adversely affect postsynaptic other non-DA neurons. Oxidative damage induces DNA instability, as well as gene expression changes, and mitochondrial stress and organic substrates oxidation ultimately produce insoluble aggregates that result in damage to the cell clearance system [19–21].

This complex neural injury mechanism network also covers all of the neurobiological mechanisms from the dopaminergic presynaptic terminus to the postsynaptic nucleus that translate METH-induced neurotransmitter dysfunction into epigenetic events that affect post expression regulation and lead to a range of neurotoxicities [21]. Epigenetic changes have been certified to take a part of an important role in regulating the gene transcriptions [21, 22]. METH may also induce epigenetic signaling cascade changes through DA-related oxidative stress effects. These include the impacts of METH act on unnatural D1- and/or D2-mediated signal transduction, REDOX-sensitive transcription factor (TF) activation, and changes in immediate early gene (IEG) expression [21].

When D1 and D2 receptor pathways are activated, activated PKA enlists mitogen-activated protein kinase (MAPK) and/or extracellular signal-regulated kinase 1/2 (ERK1/2) [23, 24]. The ERK1/2 protein may be translocated into the nucleus and then activates varieties of TFs, for example, Fos protein family, cAMP response-element binding protein (CREB), ELK-1, H3 histones, and other nuclear receptors, which all control expression of gene [23, 25–27].

METH also interacts with the abnormal activation of Glu receptors [28] to activate PLC and intracellular Ca^2+^ transducers [29, 30], thus creating a vicious cycle that further enhances oxidative stress and overactivates ERK1/2, CREB, and a large number of other TF families, including activating early growth response protein (Egr), protein 1 (AP-1),, nuclear factor activated T cells (NFAT), ELK-1, nuclear factor *κ*B (NF-*κ*B) [24]. It administrates a series of immediate early gene expressions [27, 31–34]. In addition, METH regulates the activation and/or inhibition of gene expression by affecting methylation and hydroxymethylation of DNA and/or histone acetylation and methylation [35–38]. METH impels the expression and regulation of genes encoding a sort of proteins which involved in neuronal metabolism and transcriptional regulation, as well as signal transduction between neurons. In other words, METH changed the expression levels of inflammatory cytokines, neuropeptides, and nutritional factors, as well as REDOX, endoplasmic reticulum stress, and mitochondrial stress-related events and a series of apoptotic cascade events in the brain [32–34, 39].

Oxidative stress damage caused by METH also induces neuroinflammation and toxicity in astrocytes or microglia [40, 41]. Astrocytes nourish and support neurons and, as part of the blood-brain barrier (BBB), respond to immune regulatory signals. When METH is overused, astrocyte dysfunction can lead to chronic BBB injury and subsequently spread to CNS environments dominated by inflammation, oxidative stress, and excitotoxic injury, eventually leading to neurodegeneration [42]. Microglia secrete neurotrophic factors to support neuronal function [43]. More and more evidence proved that neuroinflammation, characterized by microglial activation, plays an important role in METH-induced neurotoxicity [1]. METH induce neuron damage, especially at dopaminergic neuron endings [44]. Damaged neurons release risk-related signals, causing microglia to activate. Activated microglia produce proinflammatory substances (neurotoxins, cytokines, etc.) that exacerbate methamphetamine-related neurotoxic activity [45]. Inhibition of microglial activation and the resulting proinflammatory agents may be an important strategy for therapeutic intervention [46, 47].

METH also disrupts BBB function by inducing oxidative stress reaction in endothelial cells of the brain [48, 49]. METH-induced pathophysiological changes (high fever, low sodium, and hypertension) induce increased ROS and oxidative stress reaction in the endothelial cells of the brain and lead to increased BBB permeability and integrity destruction [49, 50]. METH gives rise to an uncontrolled RNS and ROS increasing, mitochondrial dysfunction, a release of proinflammatory cytokines, excitation of NF-*κ*B and influence endothelial physiological dysfunction and BBB damage in brain [48].

**Figure 1:**
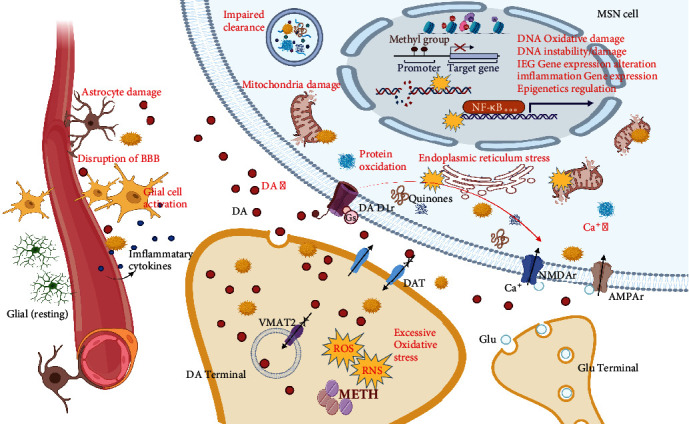
The important function of oxidative stress in the central nervous system toxicity of METH. METH enters DA energetic neurons by through the plasma membrane and prevents DA reuptake by inhibiting DAT and VMAT2, resulting in increased release of DA and excessive accumulation in the extracellular space (including synaptic clearance). Excessive quinones, ROS, and RNS accumulate in the endings of DA neurons, leading to a surge in oxidative stress levels. Extracellular ROS and RNS diffuse toward MSN neuronal and nonneuronal cells, such as the BBB neurovascular unit, astrocytes, and/or glia. Adverse effects include abnormal activation of D1 receptors; induced release of glutamate neuronal transmitters; activation of NMDA and AMPA receptors; increased intracellular Ca^2+^ concentration; further increased oxidative stress level and protein oxidation; disrupted homeostasis of the endoplasmic reticulum (ER) and mitochondria; impaired intracellular clearance system function; DNA oxidative damage and stability loss; early gene expression activation, increased inflammatory gene expression; damaged astrocytes; activation of microglia; impaired endothelial function of the BBB; increased permeability; and inflammatory factor overflow. Created with http://BioRender.com.

As shown in Figure 1, we can now map the molecular biological mechanisms of the neurotoxicity of METH. This figure clearly illustrates that these mechanisms are mostly based and centered on oxidative stress.

## 3. Importance of Nrf2 in Oxidative Stress

ROS is a generic term for a series of molecular oxygen derivatives. At the physiological level, the formation and increase of ROS play a crucial role in REDOX signaling through various posttranslational modifications, known as “oxidative stress” [51]. Moreover, other active substances are involved in the REDOX signal, such as RNS, oxidized lipids, and hydrogen sulfide [52]. In recent years, significant improvements have been made in explaining the effect of these oxidation-active substances in physiology and diseases, specifically of the cardiovascular, nervous, and immune systems, metabolic regulation, skeletal muscle aging, and even cancer [51].

ROS and REDOX form a complex, simulated REDOX network. Oxidative stress can lead to antioxidant and proinflammatory responses [52, 53]. However, this antioxidant response may not be harmful and represents the ordinary signaling paradigm, such as promoting receptor-mediated signaling by the use of ROS inactivation phosphatase [54]. Moreover, the mechanism is necessary even for truly useful stimulation, such as vascular endothelial growth factor (VEGF) [55]. Therefore, a serious reexamination of the regulation of oxidative stress-related pathways is necessary.

ROS have been identified as the driving factors about diseases previously mentioned. Studies should not only consider investigating ROS holistically but also aim to identify individual molecular targets for REDOX regulation [56]. Clinical trials have shown that nonspecific ROS elimination, using small molecules antioxidant, has not been useful in preventing the occurrence and progress of these diseases and has only shown modest benefits [57]. Thus, selectively targeted control of specific ROS-related signaling pathways provides promise for the new refined REDOX drugs. For example, the enzyme defense system is mediated by both Nrf2 and the role of trace elements, such as selenium [53].

The antioxidant system is complex regulatory network. It counteracts oxidation of ROS and RNS to maintain homeostasis of intracellular REDOX. Major antioxidants include small molecule antioxidants such as vitamin C, vitamin E, and reduced glutathione (GSH); noncatalytic antioxidant proteins, such as glutaredoxin (Grx) and thioredoxin (Trx); and superoxide dismutase (SOD), catalase, peroxidase (Prx), and glutathione peroxidase (GPx) [58].

Active oxidants are offset by a complex antioxidant defense system that is modulated by a series of pathways to ensure the oxidant response is sufficient [59]. An important oxidative and antioxidant defense signaling is REDOX signaling based on reactive cysteine mercaptans [52, 60]. The versatility and reversibility of reactive cysteine mercaptans are the basis of these REDOX signaling pathways, among which the Nrf2 signaling pathway is the most important. The modification of key cysteine mercaptans on Nrf2 is the main mechanism by which antioxidant element inducers activate Nrf2 [59, 60].

Nrf2 is a crucial transcription factor that mediates the cell oxidative stress response. It is a central regulator which maintains the homeostasis of REDOX in the cells. Nrf2 can protect cell from damage which caused through overactive oxygen species by inducing and regulating a series of antioxidant proteins expression to maintain REDOX homeostasis and to keep cells in a suitable state in the body [60].

**Figure 2:**
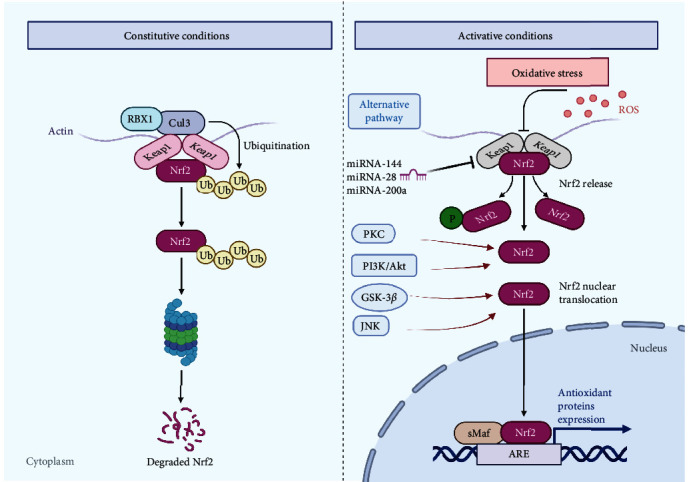
Keap1-Nrf2 pathway during constitutive and activative status. Nrf2 is adhered in the cytoplasm by Keap1 under normal physiological conditions. Keap1, as a substrate of the Cullin 3- (Cul3-) dependent E3 ubiquitin ligase complex, promotes Nrf2 ubiquitination and is rapidly hydrolyzed by the proteasome. While cells are attacked by ROS, Nrf2 dissociates from Keap1 and migrates rapidly to the nucleus. It first forms heterodimers with Maf proteins, then binds to antioxidant response elements (AREs), and regulates transcriptional activation of antioxidant oxidase gene expression. In addition, Nrf2 also has alternative activation pathways, which are regulated by phosphorylation, PKC, PI3K/Akt, and JNK to further alter the transcriptional activation of antioxidant genes. miRNA was also found to enhance Nrf2 activity by inhibiting Keap1 expression. Adapted from “Keap1-Nrf2 pathway” of http://BioRender.com (2021). Taken from https://app.biorender.com/biorender-templates.

Nrf2 protein is expressed in various body tissues (the brain, liver, kidney, spleen, heart, etc.). Its protein molecule contains seven domains of Neh1-Neh7 [59, 61]. Among them, Neh1 has a DNA binding motif. Neh3, Neh4, and Neh5 can bind to the coactivator, which is the transactivation domain of Nrf2. Neh2, Neh6, and Neh7 can all regulate the stability of Nrf2, but Neh2 is dominant since it contains two sites that can bind Kelch-like ECH-associated protein-1 (Keap1) in the cytoplasm. Named ETGE and DLG, these sites negatively regulate Nrf2 transcription activity [62]. Generally speaking, Nrf2 is settled in the cytoplasm by Keap1. Because of the Cullin 3- (Cul3-) dependent E3 ubiquitin ligase complex, Keap1 can promote Nrf2 ubiquitination and rapid degradation by the proteasome. But while cells are activated by ROS, Nrf2 dissociates from Keap1 and translocates into the nucleus rapidly. It first forms heterodimers with Maf proteins and binds to antioxidant reaction elements (AREs) to modify Nrf2 with key cysteine mercaptans. Transcriptional activation of antioxidant enzyme gene expression is regulated by Nrf2 [63]. Although it is widely accepted that the modification of one or more key cysteine residues in Keap1 (chemical addition, oxidation, nitrosylation, or glutathione) represents a possible chemobiological trigger for Nrf2 activation, clear evidence remains mysterious. Accumulated studies have shown an alternative mechanism of Nrf2 mediation, which includes Nrf2 phosphorylation by many protein kinases (GSK-3*β*, PKC, PI3K/Akt, and JNK), interactions with other protein partners (caveolin-1 and P21,) and epigenetic factors (microRNAs-144, -28, and -200a). These are potentially crucial for Nrf2 activity and thus contribute to maintaining cell homeostasis, as is shown in Figure 2 [59, 63, 64].

Nrf2 is involved in mediation of a variety of programmatic functions by regulating oxidant signals, such as inflammasome signaling, autophagy, cell apoptosis, and mitochondrial biogenesis [59, 65, 66]. Nrf2 has exhibited a variety of protective effects on toxicity and diseases, thus pointing out a new approach for drug research and development. A large number of ARE inducers have been identified, including plant chemicals and derivatives (CDDO and sulforaphane) and therapeutic agents (otipura and kinofen) [67, 68]. Additionally, ARE gene expression has been induced by Nrf2 for the treatment of diseases [59, 60]. However, due to the diversity of factors in the Nrf2 signaling pathway and the possibility of complex relationships among factors, current understanding remains very limited [67]. In addition, because clinical trials using existing antioxidants have not been very successful, the logical alternative is to activate endogenous antioxidants in the body. It may be more economical, effective, and safe to activate Nrf2, an endogenous antioxidant factor, through nondrug intervention [68].

## 4. The Important Role of Nrf2 in METH-Induced Oxidative Stress

As previously mentioned, oxidative stress from exposure to METH leads to free radical attacks on nerve cells, resulting in issues such as glial cell activation, protein misfolding, mitochondrial dysfunction, endoplasmic reticulum stress, DNA repair system damage, subsequent cell death, and BBB dysfunction [21]. Protecting neuron from oxidative damage is potential hopeful strategy for treating MUD. Nrf2 is a key transcriptional regulator that keeps REDOX homeostasis by activating the anti-inflammatory, antioxidant, and cell-protective gene expression [6]. Nrf2 can affect the function of neurons strongly by decreasing the level of reactive oxygen species, which is conducive to survival and neurogenesis [67].

Earlier studies found that methylbupropion caused Nrf2 to enter the nucleus from the cytoplasm through DA D1 reception-dependent transport in METH-induced, where it was activated and played a regulatory role [32]. METH dysregulates the REDOX state in primary rat astrocytes and midbrain neuron cultures, and in response to oxidative stress, astrocytes effectively upregulate Nrf2 transcription, expression, and nuclear translocation [69]. The expression, nuclear translocation, and binding activity of Nrf2 to the metallothionein-1 (MT-1) gene, a cysteine-rich metal binding protein with quinone quenching properties, were significantly increased in astrocytes exposed to METH, and the addition of exogenous MT-1 to neuroprotective media showed a neuroprotective effect [70]. Other studies have found that lack of Nrf2 intensifies METH-induced dopamine neuron damage and glial proliferation in the striatum. The results showed that Nrf2 had protective effects against METH-induced inflammation and oxidative stress and neuronal degeneration [71]. Using Nrf2^+/+^ and^−/−^ gene mice, the study also demonstrated that METH enhanced DNA oxidation and dopaminergic neuron ending toxicity, which was more severe in the Nrf2^−/−^ gene mice. METH can upregulate antioxidant mRNA levels and cellular protective proteins regulated by Nrf2 in the mouse brain, and Nrf2-mediated antioxidant pathways can prevent neurodegenerative effects and functional defects caused by oxidative stress in METH [72]. Studies of the differential expression of inflammatory mRNA after methamphetamine use showed that Nrf2-mediated classical pathways were enhanced in microglia [73]. It has even been reported that fetal Nrf2 deficiency can enhance oxidative DNA damage and toxicity induced by METH and that Nrf2 can reduce fetal toxicity and postnatal neurodevelopmental defects caused by METH [7].

Recent studies have shown that METH directly induces neuronal inflammation through the NF-*κ*B-dependent pathway and downregulates the transcription factor Nrf2, which is associated with several antioxidant/detoxification enzymes expression. Melatonin can inhibit activated NF-*κ*B through the activated Nrf2 pathway and enhance the antioxidant/detoxification defense pathway to play an antineuroinflammatory role [41, 74]. Melatonin also promotes the antioxidant process, regulates the expression and translocation of Nrf2, and plays a protective effect in the extra-upregulation of inflammatory cytokines and BBB inflammation induced by METH [75]. The activation effects of ARE on Nrf2 have also been reported. Lactulose can increase the expression of Nrf2, thereby inhibiting oxidative stress and neuroinflammation induced by METH and reducing neuronal autophagy and apoptosis [76, 77]. Tert-butylhydroquinone (TBHQ) attenuates METH-induced neurotoxicity through the PI3K/AKT and Nrf2/HO-1signaling pathways [78]. Trihydroxyflavanones can downregulate the neurotoxicity of METH through the PI3K/Akt/mTOR and Nrf2/heme oxygenase-1 signaling pathways [79]. Resveratrol inhibits oxidative stress and apoptosis by interacting with Keap1 and activating the Keap1-Nrf2 pathway to reduce METH-induced memory damage [80].

Elevated oxidative stress is the crucial reason of MUD. Nrf2 and its activators play a key role in those processes (as shown in Tables 1 and 2). Nondrug intervention, such as exercise, has gradually attracted attention, since it can activate endogenous antioxidants, such as Nrf2, already present in appropriate cellular sites and regulate the unbalanced REDOX state [61].

## 5. Exercise Effectively Interferes with METH Abuse

Extensive clinical studies have provided strong evidence that exercise is a cost-effective measure that can significantly decrease the burden of many diseases, including, but not confined to, cardiovascular disease, cancer, diabetes, sarcopenia, and neuropsychiatric disorders [81]. The positive effects of physical exercise on brain activity have long been studied as a nonpharmacological approach for maintaining brain health, treating neurodegenerative diseases and/or psychosis, and improving cognitive function, spatial learning and memory, anxiety, depression-like behavior, etc. [82]. Exercise has also been indicated as a possible therapy for drug addiction, either in isolated exercise-based interventions or in combination with other addiction treatment strategies [83]. The role of physical activity specific to METH addiction has also been investigated [2].

To date, limited clinical evidence supports the benefits of exercise on MUD. A total of 135 hospitalized METH-dependent subjects underwent structured aerobic and resistance training for 60 minutes three times a week for eight weeks and, after exercise, saw significantly reduced METH use [84] and improvement in METH withdrawal-related depression and anxiety symptoms [8]. A study of relationship among craving and exercise intensity of MUD found that acute exercise may have benefits for MA-related cravings in MA-dependent people. Moderate-intensity exercise perhaps have a more positive effect on MA dependence, providing prima facie evidence to establish a prescription for exercise intensity [85]. Another report based on positron emission tomography found that eight weeks of exercise training significantly improved striatal D2- and D3-receptor function in methamphetamine users [86]. Using functional near-infrared spectroscopy (fNIRS) and heart rate signal evaluation, researchers found that physical training can affect brain functional connectivity in METH-dependent subjects to a certain extent [87, 88]. Recent studies have further demonstrated that exercise can reduce depression and anxiety during withdrawal in patients with long-term METH exposure, possibly through mechanisms related to plasma BDNF and TrkB levels in peripheral blood mononuclear cells [89].

Some preclinical studies have found that wheel exercise can reduce METH self-administration or METH intake in rats [90–92], possibly by moderating METH-induced neuronal NOS [91]. Regular swimming exercise reduced METH use and improved anxiety- and depression-like behaviors in rats [93]. In terms of intervention timing, METH-induced spontaneous activity was prevented, and situational learning was promoted by motor intervention during the critical early development of mice [94]. Exercise is a crucial behavioral factor that can prevent METH-induced cerebral vascular toxicity in mice. In other words, exercise can prevent the oxidative stress of brain microvessels and the destruction of the BBB caused by METH. These evidences provide new methods for treating the toxicity of abusive drugs [95]. Another study also demonstrated that METH administration reduced tight junction (TJ) protein expression in the hippocampus and increased BBB permeability. Exercise prevents the damage by increasing TJ expression, reducing BBB integrity, and then improving neural differentiation. What is more, exercise prevents a systemic increase in METH-induced inflammatory cytokine levels. Therefore, exercise may mitigate METH-induced neurotoxicity by preventing BBB destruction in the hippocampus and associated microenvironmental changes [96]. In SD rats, forced moderate endurance exercise also inhibits METH-induced hyperactivity through the glutamate signaling pathway [97]. It is not difficult to conclude that the key link between METH-induced neurological damage and the benefits brought by exercise may be the regulation of oxidative stress mechanisms in the above [98].

## 6. The Relationship between Exercise and Oxidative Stress

In recent years, knowledge about exercise and oxidative stress has grown enormously. It has now been proved that inactive and contractile skeletal muscles produce ROS and NOS [99, 100]. Excessive exercise causes oxidative damage in contracted muscle cells [101, 102]. However, appropriate concentrations of oxidants can regulate many cellular signaling pathways and many gene expressions in the cells, including transcription, mRNA stability, and signal transduction [99, 102]. In addition, there is involvement of many products related to oxidative regulation genes including antioxidant enzymes and DNA repair proteins [99, 102]. The way that the body regulates itself to maintain this delicate oxidation-antioxidant balance is interesting.

The adverse effects of ROS have been highlighted previously, but it is more important to understand that appropriate levels of oxidative active substances are both necessary and beneficial for cellular signal transduction and epigenetic regulation [102]. Moderate exercise can regulate REDOX homeostasis in the body, thus playing a variety of health promoting roles [103]. Specifically, moderate exercise increases both the overall body's antioxidant capacity and its resistance and tolerance to oxidative stress [102].

A harmonious antioxidant system works in a coordinated way to prevent oxidative stress. Muscle cells contain a network of antioxidant defense mechanisms to reduce ROS production and increase the risk of oxidative damage [99]. Muscle fiber is rich in both enzyme-induced and nonenzyme-induced antioxidants. These antioxidants play significant roles in the cytoplasm and various organelles, respectively [99, 100, 102]. In addition, enzyme-promoted and nonenzyme-promoted antioxidants are found both outside muscle cells and within the capillary network [99]. For example, antioxidant enzymes, including superoxide dismutase, catalase, and glutathione peroxidase, convert ROS into less active molecules and prevent them from being converted into more harmful forms [101, 104]. Except for the major antioxidant enzymes described above, muscle cells contain several other important enzymes (such as those contributed by the thioredoxin, glutenodoxin, and peroxiredoxin systems) that either directly or indirectly maintain a REDOX balance [100, 102]. In addition to preventing protein oxidation, these systems can also reduce transcription factors, prevent oxidative stress, and control cell apoptosis [105]. There are many nonenzymatic antioxidants in muscle cells, including glutathione, uric acid, bilirubin, and coenzyme Q10 [106].

Oxidative stress is also associated with several other neurodegenerative diseases, such as Alzheimer's disease (AD) and Parkinson's disease (PD) [107–109]. Exercise training can slow the progression of AD and PD [110]. In rodent models, exercise has also been shown to play a beneficial role in several other brain diseases, such as ischemic stroke, spontaneous hypertension, and N-methyl-D-aspartic acid (NMDA) lesions in AD [111–113].

Several subcellular mechanisms are believed to underlie the effects of exercise on brain function, including brain damage caused by METH oxidative stress. Exercise increased the expression of antioxidants in the brain [114, 115]. Exercise can also increase other antioxidant enzymes and mitochondrial biosynthesis to improve ATP production and reducing neurodegeneration [116]. Exercise upregulates the activity of proteasome complexes and target rapamycin (mTOR) in the brain, improves cognitive performance, and reduces neurodegeneration in AD and PD [117, 118].

Exercise also induces the BDNF expression in the hippocampus, activates the downstream signaling pathway, increases CREB expression, and upregulates other transcription factors [119]. These changes can ultimately increase neuroplasticity, such as cell survival and the formation of new synapses. In addition, exercise can impact the proliferation of neuronal progenitor cells and glial cell production by regulating ROS in the hippocampus [120–122].

## 7. Status and Function of Nrf2 in Exercise Antioxidation

Studies suggested that neurons are rich in polyunsaturated fatty acids and have high metabolism and oxygen consumption and weak antioxidant capacity, so they are vulnerable to oxidative damage. However, the molecular pathogenesis of neurodegeneration is not fully understood and is speculated to be related to REDOX disorder [107]. Endogenous antioxidant mechanisms, such as the Nrf2/ARE pathway, have shown enormous potential in treating neurodegeneration and may be a worthy subject for research.

Nrf2/ARE pathway activation plays a crucial role in the response of oxidative damage to induce antioxidant defense. Nrf2 activation is a key step in adaptive antioxidant defense of exercise. Experiments have found that exercise induces Nrf2 activation [123].

During the exercise period, ROS produced directly and/or indirectly mediation, including the activation of several REDOX regulatory transcription factors, such as NF-*κ*B, heat shock factor-1 (HSF-1), activator protein-1 (AP-1), Nrf2, and PGC-1*α*. Among them, the dissociation of the Nrf2/Keap1 complex is activated by ROS to avoid degradation and, thus, maintain the Nrf2 signal. In addition, ROS can activate Nrf2 directly by cysteine modification [124]. The activation of Nrf2 leads to the enhancement of endogenous antioxidant defense ability, which enables the body to have the ability to resist oxidative stress. Nrf2 can be activated in a variety of tissues, which may be one of the important mechanisms for moderate exercise to exert systemic antioxidant effect. These effects are not only found in skeletal and/or cardiac muscle, and the activation of Nrf2 also includes the brain, liver, and kidneys [61, 66, 125].

Experiments at the cellular and animal levels also have demonstrated Nrf2-related mechanisms. In one study, C2C12 skeletal muscle cells were cultured and stimulated with electric pulses (EPS) to simulate acute exercise. Studies have demonstrated that Nrf2 is activated by ROS and that Nrf2 expression is related to the duration and intensity of excise stimulation [126]. What is more, EPS stimulated the expression of Nrf2-related antioxidant genes, while this expression was attenuated when Nrf2 transfection was knocked down by siRNA in the cells [126]. A single acute exercise session in wild-type mice resulted in Nrf2 gene expression increasing and Nrf2/ARE binding in skeletal muscle and brain tissue [127, 128], while no change of those was observed in Nrf2^−/−^ mice [129].

Reports of exercise-induced activation of Nrf2 in humans are restricted. Nowadays, study found an increased abundance of whole-cell Nrf2 protein measured in both young and elder men after acute exercise. Nuclear accumulation of Nrf2 was observed only in the young cohort, suggesting that aging is related to impaired nuclear input of Nrf2 [66]. Another cross-sectional study compared Nrf2 and Keap1 protein levels between sedentary and active older adults by using a single muscle biopsy. The result suggested that physically active adults had obvious higher levels of Nrf2 protein and a higher ratio of Nrf2 to Keap1 [130].

**Figure 3:**
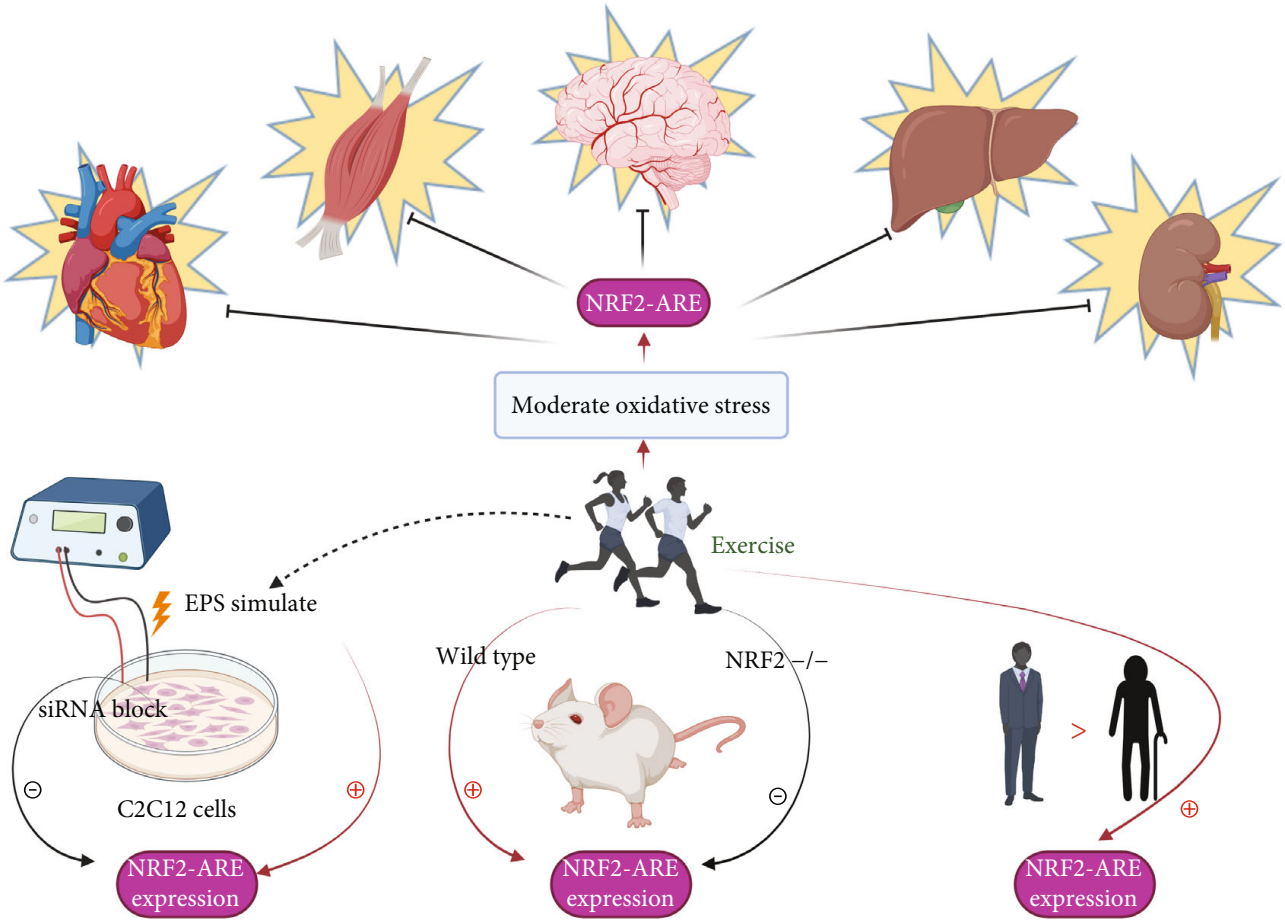
Exercise plays an important role in systemic antioxidant action through the Nrf2-ARE pathway. Nrf2 activation can occur in variety tissues. This is an important mechanism by which exercise exerts systemic antioxidant effect. These effects are not only involved in skeletal muscle and cardiac muscle but also include the brain, liver, and kidneys. Experiments at the cellular, animal, and human levels have demonstrated Nrf2-related mechanisms. Created with http://BioRender.com.

This available experimental evidence is shown in Figure 3.

## 8. Perspective of Nrf2 in Exercise Prevention and Treatment of Neurotoxicity Caused by METH Oxidative Stress

As described above, it is not difficult to conclude that long-term treatment by METH can cause oxidative stress, lipid peroxidation, endoplasmic reticulum stress, mitochondrial dysfunction, and nerve toxicity [3]. Additionally, toxic effects of excitement and nerve inflammation on cells, both internally and externally, can lead to neuronal apoptosis, abnormal activation of glial cells, and BBB dysfunction, eventually leading to whole-brain nerve toxicity and dysfunction [42]. Protecting neuron from oxidative damage is a promising strategy for treating MUD. Since clinical trials about antioxidants exogenous application are not ideal, it may be more economical, effective, rational, and safe to activate endogenous antioxidants through nonpharmaceutical interventions [48].

Nrf2 is a major transcriptional regulatory factor that keeps cellular REDOX equilibrium through activating antioxidant and cell-protective genes expression by means of endogenous means [51, 60]. Nrf2 can affect the function of neurons strongly by reducing the level of ROS, which is conducive to survival and neurogenesis. Both preclinical and clinical studies reported that Nrf2 had a protective effect on METH-induced oxidative stress and inflammation, even neuronal degeneration [41]. The Nrf2-mediated antioxidant pathway can prevent neurodegenerative effects and functional defects caused by oxidative stress in METH [60, 97].

**Figure 4:**
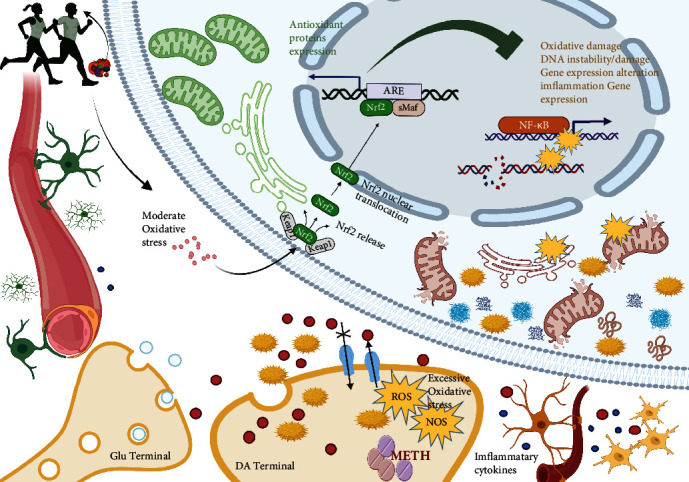
Exercise exerts endogenous antioxidant regulation through the Nrf2 signaling pathway to fight brain oxidative stress injury caused by METH. Exercise has great potential to play an endogenous antioxidant regulatory role through the Nrf2 signaling pathway to decrease harms caused by methamphetamine-induced oxidative stress. However, more experimental evidence is needed to support this idea. Further exploration of the different effects of various parameters of an exercise intervention on Nrf2 signaling pathway intervention (such as exercise mode, time, and intensity) is needed. Whether there is a synergistic effect between exercise and phytonutrient Nrf2 activators is worth further investigation. Created with http://BioRender.com.

Nondrug intervention, such as exercise, can activate endogenous antioxidants already present in appropriate cellular sites and also regulate the unbalanced REDOX state, an idea that is gradually attracting attention [65, 96]. The exercise-induced oxidative stress result is still a controversial problem. It appears that exercise-induced ROS production may have both positive and negative consequences [127]. High levels of ROS production during exercise may lead to the destruction of macromolecular structures, while moderate levels of ROS production promote the positive physiological adaptation of active skeletal muscle. The prevailing view is that moderate exercise can activate endogenous antioxidant systems, such as Nrf2, and induce endogenous antioxidant defense [66, 102]. Therefore, we contend that exercise has great hope to play an endogenous antioxidant regulatory role through the Nrf2 signaling pathway to decrease the harm caused by methamphetamine-induced oxidative stress, including methamphetamine-induced, oxidative stress brain damage; this scenario is shown in Figure 4.

Unfortunately, relevant studies are limited, and currently, only one report has suggested the potential role of Nrf2 in oxidative stress injury of METH-induced by exercise. Acute exposure to METH induces severe oxidative stress and destruction of BBB. Endurance exercise training can prevent these effects caused by METH, among which the increase in Nrf2 phosphorylation induced by exercise is one of the involved antioxidant mechanisms [95]. Further exploration of the underlying mechanisms is needed and will be of interest in the future. In particular, exploration of how to conduct “cross-talk” between mild oxidative stress effects of skeletal muscle and brain neurons to produce antioxidant protection effects is of interest. This topic will be beneficial to the further exploration of the mechanisms of effective exercise intervention in major nervous system diseases, including METH brain injury.

As with other neurodegenerative diseases in which oxidative stress is the core induction mechanism, the following issues should be addressed in the treatment of METH oxidative stress brain injury events. Although some animal studies have shown that Nrf2 signaling increases depending on exercise duration [120, 122], some questions remain. Almost no existing researches have explored the effect of exercise intensity on the Nrf2 signal pathway. Similarly, there are few studies investigating the response of Nrf2 signaling to traditional resistance training. Moreover, this research did not address the Nrf2 signal itself, only measuring changes about Nrf2 gene expression. More explorations need to clarify whether the resistance training could activate Nrf2 signaling [66, 120].

In addition, it should be stressed that although the evidence clearly supports that exercise induced by activation of Nrf2 is a feasible method of endogenous antioxidant defense, exogenous antioxidant supplements can also reduce free radicals and promote cell oxidation-reduction equilibrium; people are always interested in whether exogenous supplementation or drugs may be used to achieve this goal [131]. It has been documented that the influence of exercise can be restrained by the simultaneous supplement of antioxidants but reinforced by the simultaneous administration of plant-derived Nrf2 activator [66, 78]. Combined with existing Nrf2 activators, exercise may have a potential synergistic effect to further improve endogenous REDOX dynamic balance [66]. Therefore, whether this synergistic effect between exercise and phytonutrient Nrf2 activators exists is worth further investigation.

## Figures and Tables

**Table 1:** The role of NRF2 in the oxidative stress induced by METH.

Species	Research object	Mechanism	Consequence	Ref.
Rats	Striatum	Nrf2 enter the nucleus through DAD1 reception-dependent transport	Activated and played its regulatory role on endoplasmic reticulum stress-related molecular events	[32]
Rat	Primary astrocytes and midbrain neuron	Astrocytes effectively upregulated Nrf2 transcription, expression, and nuclear translocation	Response to oxidative stress	[69]
Mouse	Dopaminergic neurons	Astrocytes derived expression, nuclear translocation, and binding activity of Nrf2 to metallothionein-1 (MT-1) gene significantly increased	Quinone quenching and neuroprotective effect	[70]
Nrf2 +/+ and -/- gene mouse	Dopaminergic neurons	Nrf2 deficiency	Exacerbates METH induced dopamine neuron damage and glial proliferation; DNA oxidation and dopaminergic nerve endings toxicity more severe	[71]
Nrf2 +/+ and -/- gene mouse	Dopaminergic neurons	Upregulate the mRNA levels of antioxidants and cellular protective proteins regulated by Nrf2	Prevent neurodegenerative effects and functional defects caused by oxidative stress of METH	[72]
Sprague-Dawley rat	Striatum and prefrontal cortex	Nrf2-mediated classical pathways were enhanced in microglias after METH exposure	Differential expression of inflammatory mRNA	[73]
Fetal	Brain	Nrf2 deficiency	Enhance oxidative DNA damage, toxicity, and postnatal neurodevelopmental deficits induced by METH	[7]
Human	Dopamine SH-SY5Y cell lines	Through the NF-*κ*B dependent pathway, down-regulates Nrf2 associated with the expression of several antioxidant/detoxification enzymes	Induces neuronal inflammation	[74]

**Table 2:** The role of NRF2 stimulators in the oxidative stress induced by METH.

Stimulator	Species	Research object	Mechanism	Consequence	Ref.
Melatonin	Human	Glioma cell line	Through NF-kappaB and Nrf2 pathways	Protects methamphetamine-induced neuroinflammation	[41]
	Human	Dopamine SH-SY5Y cell lines	Through the NF-*κ*B dependent pathway, upregulates Nrf2 associated with the expression of several antioxidant/detoxification enzymes	Reduces neuronal inflammation	[74]
	Rat	BBB	Promotes the antioxidant process, regulates the expression and translocation of Nrf2	Plays a protective role in the over-upregulation of proinflammatory cytokines and BBB inflammation induced by METH	[75]
Lactulose	Rat	Striatum	Through suppressing oxidative stress and neuroinflammation	Alleviated METH-induced neurotoxicity	[76]
	Rat	Striatum	Alleviating autophagy, stabilizing antioxidant system and suppressing apoptosis	Attenuates METH-induced neurotoxicity	[77]
TBHQ	Rat	VTA	Through the Nrf2/HO-1 and PI3K/AKT signaling pathways	Attenuates neurotoxicity induced by METH	[78]
6,7,4′-Trihydroxyflavanone	Human	SH-SY5Y cells	Nrf2/heme oxyganase-1 and PI3K/Akt/mTOR signaling pathways	Mitigates METH-induced neurotoxicity	[79]
Resveratrol	Mice	Hippocampal	Inhibition of oxidative stress and apoptosis	Attenuates methamphetamine-induced memory impairment	[80]

## Data Availability

The data used to support the findings of this study are included within the article.
